# Albuminuria and Eclampsia during Pregnancy

**Published:** 1882-07

**Authors:** 


### ALBUMINURIA AND ECLAMPSIA DURING PREGNANCY.

In a communication upon the above subject, published in the *Zeitschrift für Geburtshülfe und Gynäkologie*, Dr. Ingerslev, of Copenhagen, brings forward some new statistical facts which are of importance. He is opposed to those who hold that the occurrence of albuminuria in pregnancy is explained by pressure on the renal veins. He shows by comparing statistics from different authors, the great divergence of statements as to the frequency of albuminuria during pregnancy, the wide differences being no doubt partly accidental, but also dependent upon the period of pregnancy at which the examination was made (some authors having included cases in which the urine was not examined till labor had begun), and upon the care which was taken to ascertain the source of the albumen. Dr. Ingerslev gives six hundred cases, in which the urine was carefully drawn off with a catheter, so as to avoid any admixture of other secretions. In twenty-nine of these, or 4.8 per cent, albumen was present. In seven microscopical examination revealed casts. Of these six hundred, three hundred and forty-eight were pregnant for the first time. As to the period of pregnancy, five were in the fourth month, albumen being present once; thirteen in the sixth month, in one albumen being present; thirty-six in the seventh month, none of them showing albuminuria; one hundred and seventy in the eighth month, albumen being present in nine; two hundred and eighty-one in the ninth month, with albuminuria in thirteen; and ninety-five in the tenth month, albumen being present in five. Of the six hundred pregnant women, more or less edema of the lower extremities was present in ninety-six. Of the twenty-nine with albuminuria, edema was present in seven. In five there was hydramnios, and five were twin pregnancies, but in none of these was there albumen. In one there was chronic heart disease (mitral regurgitation) with albumen in the urine.

The next point upon which Dr. Ingerslev contributes some facts is as to the persistence of albuminuria after delivery. Out of thirty-six cases in which albuminuria was present during pregnancy, eight died, fourteen recovered, and fourteen were lost sight of while albumen was still present. Of the fourteen who recovered, in seven the albuminuria lasted five days; in four, fourteen days; in two, thirty days; in one, sixty days after delivery. Of those in whom albuminuria continued as long as they were under observation, in three it was ascertained to persist twenty days; in five, one month and a half to two months; in two, three months; in one, five months; in two, six months; in one, seven months after labor. It follows, therefore, that in cases of albuminuria with pregnancy, the prognosis as to ultimate recovery should be guarded.

With regard to the effect of the process of labor in producing albuminuria, Dr. Ingerslev gives one hundred and fifty-three cases in which the urine was examined during labor. In fifty of them albumen was present, or about 32 per cent. Of these fifty, forty-six were also examined during pregnancy, but in only fifteen of them was albumen then present. In forty-one out of the fifty the subsequent course was ascertained. In eight the albumen had disappeared the next day; in twenty-five on the second day ; in one on the fourth ; in one on the seventh ; in one on the ninth ; and in one on the thirteenth day. In four cases chronic cystitis followed. In brief, in 80.5 per cent the urine became normal in forty-eight hours.

As to the connection between eclampsia and albuminuria, out of one hundred cases of eclampsia in the Copenhagen Lying-in Hospital, the urine was examined in seventy-seven, and in seventy-one albumen was present, in six being absent. Out of the seventy-one, in twenty general anasarca was present ; edema only of the lower extremities in thirty-six ; and in fifteen no edema. In thirteen cases albuminuria was known to have preceded the eclampsia ; in the remainder it was not detected till simultaneously with, or after, the convulsions. As to the course of the albuminuria, in twenty-six it disappeared within five days, or 40 per cent ; in thirty-nine, or 60.9 per cent, within fourteen days. The view as to the pathology of puerperal albuminuria and eclampsia that Dr. Ingerslev adopts is that it is the manifestation of an especially acute nephritis, and that the albuminuria, eclampsia, and nephritis are co-ordinate phenomena, results of a vasomotor reflex neurosis. This is a view which it is difficult to controvert, for there is scarcely any acute disease which is not accompanied by some alteration in the action of the vasomotor system, and therefore might not be called a vasomotor neurosis. —*Medical Times and Gazette.*

